# Advanced Clinical Neonatal Nursing Students’ Transfer of Performance: From Skills Training With Real-Time Feedback on Ventilation to a Simulated Neonatal Resuscitation Scenario

**DOI:** 10.3389/fped.2022.866775

**Published:** 2022-04-18

**Authors:** Irene Rød, Anna-Kristi Jørstad, Hanne Aagaard, Arild Rønnestad, Anne Lee Solevåg

**Affiliations:** ^1^Department of Master and Postgraduate Education, Lovisenberg Diaconal University College, Oslo, Norway; ^2^Faculty of Medicine, University of Oslo, Oslo, Norway; ^3^Department of Neonatal Intensive Care, Division of Pediatric and Adolescent Medicine, Oslo University Hospital, Rikshospitalet, Oslo, Norway

**Keywords:** advanced neonatal nursing education, resuscitation, real-time feedback, simulation, bag-mask ventilation

## Abstract

**Background:**

Advanced clinical neonatal nurses are expected to have technical skills including bag-mask ventilation. Previous studies on neonatal bag-mask ventilation skills training focus largely on medical students and/or physicians. The aim of this study was to investigate whether advanced clinical neonatal nursing students’ bag-mask ventilation training with real-time feedback resulted in transfer of bag-mask ventilation performance to a simulated setting without feedback on ventilation.

**Materials and Methods:**

Students in advanced clinical neonatal nursing practiced bag-mask ventilation on a premature manikin (Premature Anne, Laerdal Medical, Stavanger, Norway) during skills training. A flow sensor (Neo Training, Monivent AB, Gothenburg, Sweden) was placed between the facemask and the self-inflating bag (Laerdal Medical), and visual feedback on mask leak (%), expiratory tidal volume (VT_*e*_ in ml/kg), ventilation rate and inflation pressure was provided. Two months later, the students participated in a simulated neonatal resuscitation scenario. The same variables were recorded, but not fed back to the students. We compared ventilation data from skills- and simulation training. A structured questionnaire was used to investigate the students’ self-perceived neonatal ventilation competence before and after the skills- and simulation training.

**Results:**

Mask leakage and ventilation rate was higher, and VT_*e*_ lower and highly variable in the simulated scenario compared with skills training (all *p* < 0.001). There was no statistically significant difference in inflation pressure (*p* = 0.92). The fraction of ventilations with VT_*e*_ within the target range was lower during simulation (21%) compared to skills training (30%) (*p* < 0.001). There was no difference in the students’ self-perceived competence in bag-mask ventilation before vs. after skills- and simulation training.

**Conclusion:**

Skills training with real-time feedback on mask leak, ventilation rate, tidal volume, and inflation pressure did not result in objective or subjective improvements in bag-mask ventilation in a simulated neonatal resuscitation situation. Incorrect VT_*e*_ delivery was common even when feedback was provided. It would be of interest to study whether more frequent training, and training both with and without feedback, could improve transfer of performance to a simulated resuscitation setting.

## Introduction

When a newborn infant needs stabilization and resuscitation, the advanced clinical neonatal nurse participates in the team. There are three intrinsic elements of human performance in these situations: 1. technical skills; and non-technical, i.e., 2. Cognitive, and 3. behavioral skills. According to the “sociotechnical systems” theory, these elements are to some extent inseparable from each other (1–4). A concept related to the sociotechnical systems theory is the “human factor” aspect, which can be attributed to the individual, team, or the way individuals interact with the environment (5, 6). At the individual level, extensively studied human factors include cognition, fatigue, and physical ability (7). Healthcare professionals’ performance in neonatal resuscitation depends on cognitive- and technical skills, as well as human factors (8).

Although, airway management is often the responsibility of physicians and respiratory therapists, the advanced clinical neonatal nurse is expected and required to have the knowledge, technical and non-technical skills, and execute physical and mental tasks, actions, and functions as a part of the resuscitation team. However, studies have shown that even experienced healthcare professionals have problems performing correct bag-mask ventilation (9, 10). Challenges include mask leakage, too low or high ventilation rate, and variable delivery of volume and pressure to the lungs (9). Both under- and over-delivery of volumes and pressures to the lungs may be harmful, causing prolonged hypoxia and bradycardia, and lung overdistension (volu- and barotrauma) and hypocapnia, respectively (11). The risk of negative long-term consequences of inappropriate ventilation may be particularly pronounced in premature infants because their lungs are immature and surfactant deficient, and their brains more vulnerable to fluctuations in blood carbon dioxide partial pressure (9, 11).

The International Liaison Committee on Resuscitation recommends programs that include simulation training with feedback from different sources including devices such as respiratory monitoring devices (12). Real-time feedback may prompt healthcare professionals to focusing on reducing mask leak and optimizing ventilation rate, tidal volume and inflation pressure, as well as provide faster learning (13) and improve the quality of resuscitation (14). During simulated neonatal cardiopulmonary resuscitation, respiratory monitoring devices, and verbal feedback were helpful methods to reduce mask leak and increase tidal volume significantly (15). Feedback on expired tidal volume (VT_*e*_) and ventilation pressure helps to identify the need to make adjustments in the mask position or in the positioning of the infant, and may also aid the identification of airway obstruction and changes in lung compliance (14). A recent randomized crossover simulation study found that real-time feedback improved the quality of pediatric resuscitation performed by medical students, neonatal fellows and consultants, and *one* nurse (10), reinforcing that studies on bag-mask ventilation skills training focus largely on medical students and physicians.

Thus, the objective of this study was to investigate whether bag-mask ventilation skills training with real-time feedback on mask leak, delivered tidal volume, ventilation rate, and inflation pressure, enabled students in advanced clinical neonatal nursing to perform high-quality bag-mask ventilation in a simulated neonatal resuscitation scenario.

## Materials and Methods

### Context and Setting

Students in the master’s degree program normally participate in simulation training every half-term, but due to the Covid pandemic, the first-term simulation training was replaced with an online training case, using a video-recorded simulated scenario. This present project was conducted in the second half-term of the master’s degree program in advanced clinical neonatal nursing. Skills and simulation training took place in the simulation room at Lovisenberg Diaconal University College (LDUC), Oslo, Norway.

### Participants and Ethics

The requirement for admission to the master’s degree program is a completed Bachelor’s degree in nursing. Additionally, students must have at least 2 years of full-time postgraduate nursing experience, including minimum 1 year of full-time experience relevant to the program. Norwegian neonatal intensive care units (NICUs) are classified based on the level of medical treatment and neonatal care that they provide (16). The students in this study worked in category 3a units (cares for sick term infants and premature infants with gestational ages ≥ 28 + 0, usually > 1,200 grams), 3b units (cares for sick term infants and premature infants with gestational ages ≥ 26 + 0, usually > 900 grams) and 3c units (cares for sick term infants and premature infants with gestational ages ≥ 23 + 0).

The project was approved by the head of the faculty at LDUC and the Norwegian Center for Research Data (NSD). All students at the master’s degree program in advanced clinical neonatal nursing received oral information and a written invitation to participate in the study, and the participating students gave their written consent.

### Monivent Neo Training

The Neo Training system (Monivent AB, Gothenburg, Sweden) is designed to support neonatal ventilation skills training, and improve and maintain manual ventilation skills (17). Monivent Neo Training measures flow via a sensor module connected as a spacer between the manual ventilation device and a facemask. Wireless data transmission improves realism by eliminating the need for cables or pipes. The sensor module provides continuous measurements that are fed back as ventilation parameters including mask leakage, tidal volume, ventilation rate, and inflation pressure. Real-time feedback is displayed numerically and graphically on an external monitor/tablet. A color indicator on the monitor/tablet and on the sensormodule shows whether the *tidal volumes* are within a specified target range. The color indicator thus serves as a guide to making adjustments to improve ventilation.

### Skills Training

In August 2021, the students at the master’s degree program (*n* = 24) participated in training on technical skills related to bag-mask ventilation of a term and a premature manikin.

In alignment with the International Nursing Association for Clinical Simulation and Learning (INACSL) (18), learning objectives were aligned with the curriculum for the master’s program in advanced clinical neonatal nursing and based on the theory of deliberate practice. Deliberate practice is a training approach where learners are given: a discrete goal to achieve, immediate feedback on their performance, and ample time for repetition to improve performance (19).

Lectures were given in advance of the skills training and focused on the guidelines for resuscitation, and theoretical and practical perspectives on neonatal ventilation. The lectures addressed how to place the face mask, definitions of effective ventilation (tidal volume 4–6 ml/kg and rate 30 min^–1^), and ventilation corrective actions, e.g., establishment and maintenance of open airways, repositioning of the baby’s head to secure a neutral position, and chin-jaw lifts to enlarge the pharynx. The ventilation skills training was organized in two sessions. In the first session, the students practiced bag-mask ventilation on a term manikin (Baby Anne, Laerdal Medical, Stavanger, Norway) to ensure that they managed basic ventilation skills prior to the commencement of the study data acquisition. This manikin has a realistic airway resistance that allows students to learn the important techniques of opening the airways and chin-jaw lifting (20). In groups of three, the students, in turn, practiced bag-mask ventilation with verbal guidance from a trained facilitator.

In the second session, i.e., the study baseline session, the students practiced one-by-one on a premature manikin (Premature Anne, Laerdal Medical, Stavanger, Norway). Each student ventilated the manikin for 3 min. During this session, they got visual real-time feedback on ventilation using the Monivent Neo Training system, supplemented by verbal feedback from a trained facilitator.

### Simulation Training

In October 2021, full-scale simulations were performed in accordance with recommendations from the INACSL with briefing, simulation, debriefing and evaluation (18). A trained simulation facilitator was responsible for all four phases of the simulation. The objectives and scenario were presented to the students prior to the simulation training. Each simulation session lasted approximately 75 min; 10 min were used to prepare and present the students to the manikin and simulation room, the simulation scenario lasted 20–25 min, followed by a 30–40 min debriefing. The resuscitation teams in the simulated scenarios consisted of three students (*n* = 24) and one physician. In each team, one student was assigned the main nurse role with responsibility for airway management. The other two students were handling medications, prepared equipment for intubation, etc. All teams simulated the scenario twice, with a change of tasks and roles between the two scenarios. The time schedule allowed for ten students to be randomly selected to take the main nurse role of managing the airways in a scenario. The premature manikin (Premature Anne, Laerdal Medical, Stavanger, Norway) was used and the same ventilation variables as in the second session of the skills training (premature manikin) were recorded with the Monivent Neo training system. However, the students did not receive feedback on the ventilation parameters.

### Monivent Data Collection and Recording

Testing of the setup with support from Monivent AB revealed no internal air leak in the Premature Anne manikin airways. The target VT_*e*_ was 4–6 ml/kg and set according to a 750 g infant (Premature Anne, Laerdal Medical). A flow sensor (Neo Training, Monivent AB) was placed between the facemask (Monivent AB) and a self-inflating bag (Laerdal Silicone Resuscitator, Laerdal Medical) to measure and display on a tablet (iPad, Apple Inc., Cupertino, CA, United States), graphically and numerically mask leakage (%), expiratory tidal volume (VT_*e*_ in ml/kg), ventilation rate (min^–1^) and inflation pressure (cm H_2_O) in real-time. The tablet wirelessly stored the variables for later analysis.

### Questionnaire 1

After skills- and simulation training had both been completed, a structured Likert-scale questionnaire was used to investigate the students’ self-perceived competence in bag-mask ventilation before vs. after skills- and simulation training. In the questionnaire, the students also reported in which category NICU they were employed, whether they had performed neonatal ventilation in clinical practice, and the number of years of clinical work experience.

### Questionnaire 2

To shed light on the results, a *post-hoc* questionnaire was distributed to investigate the students’ preferred ventilation device, whether they were used to being in charge of airway management, and about their perceived stress in the simulation training.

### Data Handling and Statistical Analyses

We tested for differences in the fraction of correct ventilations (VT_*e*_) between sessions (skills- or simulation training) by making cross tabulations. To examine the direction of potential differences, we made box and whisker plots and bar charts. Continuous variables were compared between sessions with Mann-Whitney *U* non-parametric test. Categorical variables are presented as numbers with percent and continuous variables as median with interquartile range (IQR). *P*-values are 2-sided and significance level < 0.05. Statistical analyses were performed with IBM SPSS 27 for Mac (IBM Corporation, Armonk, NY, United States).

## Results

Data were registered from the 10 students who performed bag-mask ventilation both in the skills training and during simulation. These students had median (IQR) years of clinical experience of 7 (4–11) years, ranging from 3 to 16 years. One student worked in a category 3a NICU, two students in a category 3b NICU, and seven students in a category 3c NICU. Except for the student from the 3a NICU and one student from a 3c NICU, all students reported having performed neonatal ventilation in clinical practice. Eight students answered the *post-hoc* questionnaire 2. Five students preferred the T-piece, two preferred a bag, and one had no preferred device for manual ventilations. Three students were familiar with being responsible for airway management, whereas five students were not used to having this role. The simulation training was perceived as being stressful, but for most (*n* = 6), experienced as positive stress, e.g., helping them to stay focused.

### Summary Results All Students Pooled

We analyzed 4.166 ventilations, 876 during skills training and 3.290 during simulation.

In the skills training, 52% of ventilations had a higher than recommended VT_*e*,_ and 18% lower than recommended. In the simulation training, 31% of ventilations had a higher than recommended VT_*e*,_ and 48% lower than recommended. The fraction of correct ventilations (30% versus 21%) was significantly different between skills and simulation training (*p* < 0.001).

**FIGURE 1:**
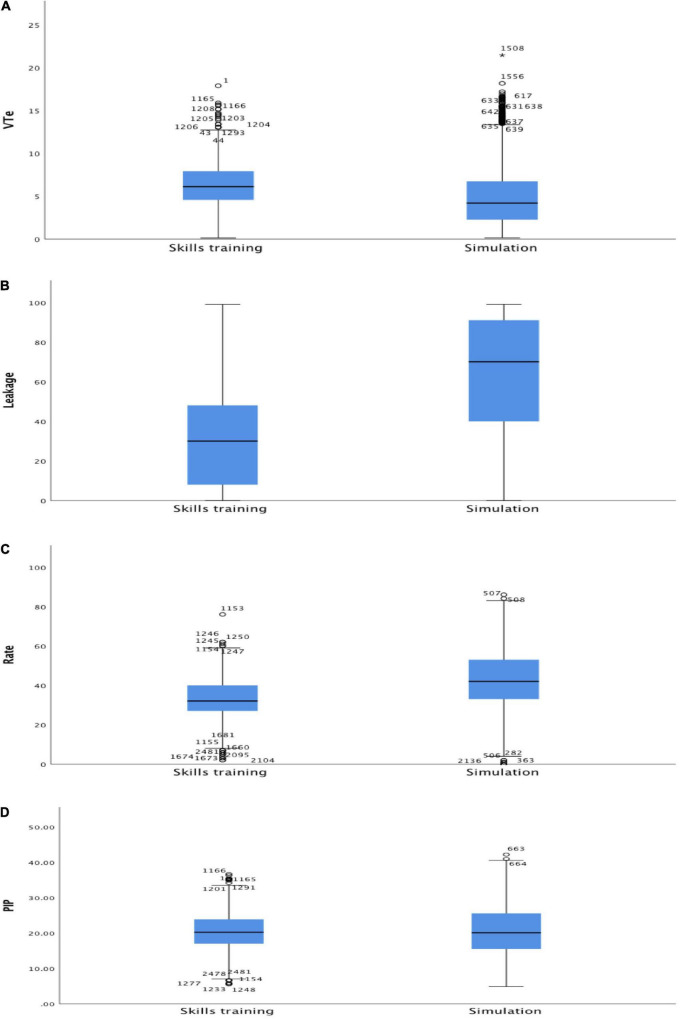
Box plots all students combined of **(A)** expiratory tidal volume (VT_*e*_, ml/kg), **(B)** leakage (%), **(C)** ventilation rate (min^–1^), and **(D)** positive inspiratory pressure (PIP, cmH_2_O) during skills and simulation training. Within each box, the horizontal black line represents the median value; boxes extend from the 25th to the 75th percentile; while the whiskers represent the minimum and maximum values, respectively.

Figure 1 presents ventilation parameters during skills- vs. simulation training. Leakage was 30 (8–48) and 70 (40–91)% (*p* < 0.001), respectively; VT_*e*_ was 6 (5–8) and 4 (2–7) ml/kg (*p* < 0.001), ventilation rate 32 (27–40) and 42 (33–53) min^–1^ (*p* < 0.001), and inflation pressure 20 (17–24) and 20 (15–25) cmH_2_O (*p* = 0.92), respectively.

### Individual Student Results

**FIGURE 2:**
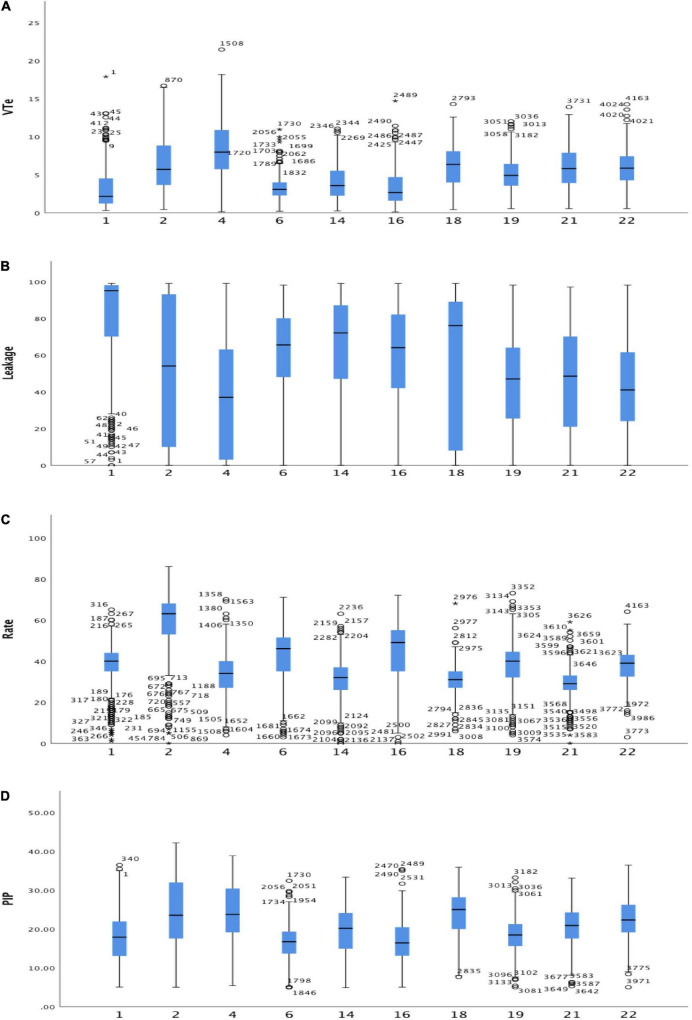
Box plots individual students, skills training and simulation combined of **(A)** expiratory tidal volume (VT_*e*_, ml/kg), **(B)** leakage (%), **(C)** ventilation rate (min^–1^), and **(D)** positive inspiratory pressure (PIP, cmH_2_O). Within each box, the horizontal black line represents the median value; boxes extend from the 25th to the 75th percentile; while the whiskers represent the minimum and maximum values, respectively.

**FIGURE 3:**
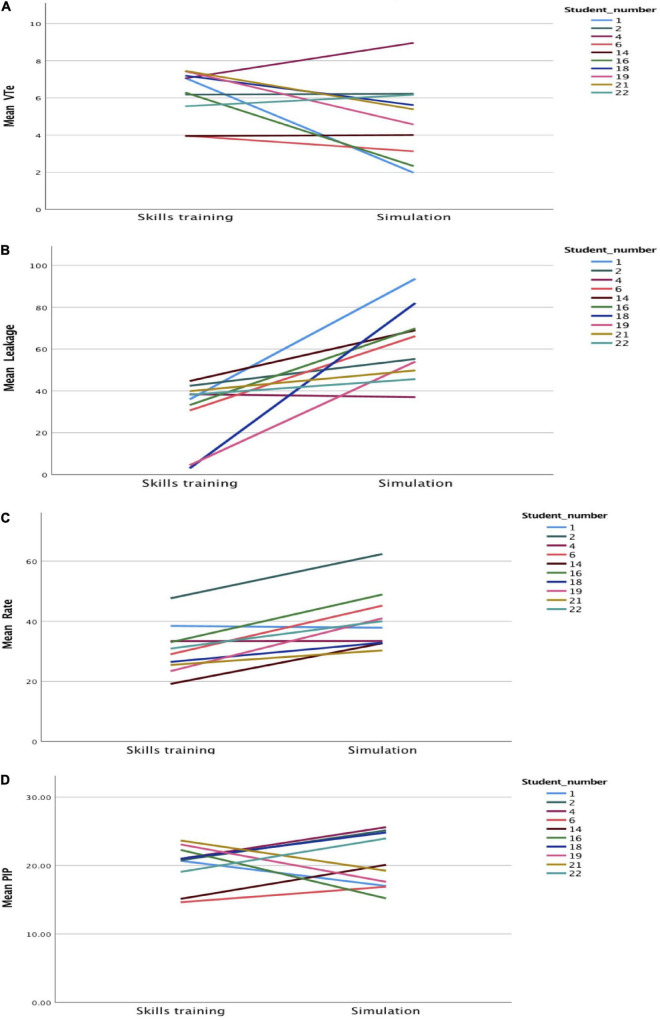
Spaghetti plots individual students of **(A)** expiratory tidal volume (VT_*e*_, ml/kg), **(B)** leakage (%), **(C)** ventilation rate (min^–1^), and **(D)** positive inspiratory pressure (PIP, cmH_2_O) during skills and simulation training.

Figures 2, 3 present individual student results. One student (#4) had similar results for mask leak and ventilation rate during skills training and simulation. The other students had results consistent with the summary results, i.e., a higher leakage and ventilation rate, and lower VT_*e*_ in the simulation compared with skills training.

### Questionnaires

**FIGURE 4:**
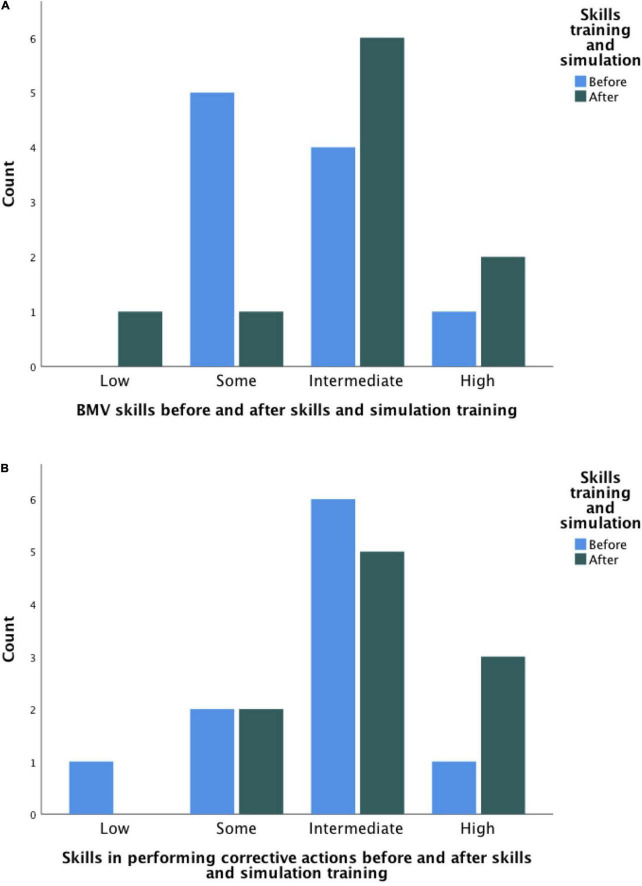
Bar chart of students’ self-assessed skills in **(A)** bag-mask ventilation (BMV) and **(B)** ventilation corrective actions before (blue) and after (green) skills and simulation training.

The results of questionnaire 1 are presented in Figure 4. One student perceived his/her bag-mask ventilation and corrective action skills as being lower after skills- and simulation training but expressed that the lectures, skills training, and simulation contributed to an increased level of competence. In the remaining questionnaires, there was a slight improvement in how the students assessed their own skills in bag-mask ventilation and corrective actions, with more students rating their skills as “high” after skills- and simulation training. However, the difference was not significant (*p* = 0.22 and *p* = 0.55 for bag-mask ventilation skills and ventilation corrective actions, respectively).

## Discussion

In previous studies, results from real-time feedback have either been masked or made visible to the participants during ventilation (10, 14). In this study, the participants trained their ventilation skills with visible real-time feedback, followed by a simulation without visible feedback. The results showed that, almost uniformly, advanced clinical neonatal nursing students had a higher mask leak and ventilated at a higher rate during simulation compared to skills training. Despite a high mask leakage, median VT_*e*_ was within the recommended range both during skills- and simulation training. However, VT_*e*_ was highly variable with a high rate of both under- and over-inflation. The inflation pressure was quite consistent and within the recommended range in both the skills and simulation training.

Our results are in agreement with Gomo et al. (21) who found that mask leakage itself did not impair tidal volume delivery. We found a median mask leak of 30% with feedback and 70% without feedback, but a VT_*e*_ within the recommended range. Gomo et al. (21) speculated that the leak is not constant but dynamic, which suggests that some variation in leakage and VT_*e*_ can be tolerated. Notably, in our study, the fraction of correct VT_*e*_’s was very low both in the skills training and simulation.

Despite our students’ *post-hoc*-reporting of “positive stress,” a higher ventilation rate may reflect a negative stress-response during simulation. Resuscitation situations often cause stress among the involved staff (22), and to relieve the stress in such acute complex situations, theoretical knowledge and practical skills are important (8). Our students participated in lectures to provide them with theoretical knowledge, and practical skills training. Unfortunately, these learning activities did not result in the expected learning outcomes of high-quality ventilation in a simulated scenario.

It is the responsibility of both the individual healthcare professional, educational institutions and the NICU leadership that knowledge of and skills in providing safe and effective ventilation are repeatedly trained and tested. The results from this study contrast with studies that found that training with real-time feedback improved ventilation performance (10, 14), and in the following we discuss potential causes for this discrepancy.

### The Time Between Skills Training and Simulation

Anderson et al. (23) aimed to find the optimal frequency of resuscitation skills training for learning and retention, and found that the time between training sessions negatively correlated with nurses’ cardiopulmonary resuscitation (CPR) performance. They concluded that brief, but frequent training on a manikin improved performance. The monthly frequency of training scored higher on performance than training every third, sixth, and twelfth month (23). Although the study investigated CPR skills on adult, not premature manikins, it is likely that the results are transferable since both adult and neonatal resuscitation skills need to be maintained to be used in similar stressful situations. This is further supported by van Vonderen et al. (24) who demonstrated that 2 min daily ventilation training improved NICU staff’s ventilation skills. The study contended that a 2-min training session is manageable for any unit if it is planned and prioritized (24).

### Clinical Experience and Role

Our participants were adult learners and had a median (IQR) year of clinical experience of 7 (4–11) years, but only 8 out of 10 reported to have performed neonatal ventilation in clinical practice. According to Mumma et al. (25), an *expert provider* has more than 8 years of professional experience from working in a critical environment, while a *novice* has no more than 2 years of professional experience. The student who had consistent results for mask leakage and ventilation rate during skills training and simulation had 5 years of clinical experience, no hands-on experience with clinical neonatal ventilation, and scored “to some extent” on self-perceived competence before skills- and simulation training. We may only speculate how this student managed to perform well in the simulation, e.g., by observing manikin chest rise and by utilizing the lessons learned from the skills training. Another explanation may be that this student has a higher appraisal of her stress-coping ability, and accordingly, lower perceived stress than the other students, this will be discussed later.

Except for the student from 3a NICU and one student from a 3c NICU, all students reported having performed neonatal ventilation in clinical practice. In clinical practice, nurses must be properly trained in the early identification of clinical deterioration (26) and must be prepared to initiate stabilizing measures including bag-mask ventilation as physicians are often not immediately available (27). Despite the students’ experience from level 3 NICUs, it can still be assumed that this was an unfamiliar role for the students. The *post-hoc* questionnaire confirms our assumptions that the simulated scenario may have caused a different stress response in our nursing students than the pediatric trainees in the study by Lizotte et al. (22).

### Stress and Non-technical Skills

Having an unfamiliar role in the simulated setting may contribute to participant stress. Lizotte et al. (22) found that resuscitation simulations caused both anticipatory and participatory stress in pediatric residents, measured by salivary cortisol (objective stress) and a questionnaire (subjective stress). Anticipatory stress is described as stress that occurs prior to a simulation. Participatory stress is the stress experienced during the simulation (22). Surprisingly, neither objective, nor subjective stress interfered with the participants’ performance (22). If the result of that study is transferable to our participants, stress may not be the main reason for the suboptimal ventilation performance during the simulation.

All 10 students had the same lecture and skills training 8 weeks before the simulation, and all of them were experienced nurses working in relatively high-acuity level 3 NICUs. However, some individual differences will naturally appear in a group. These characteristics can be understood as non-technical skills and may have contributed to the differences in performance. The students in this study confirmed that simulation training is a stressful situation but stated that they became more focused on the tasks and managed to stay calm even though they were stressed. Non-technical skills include cognitive and social skills that complement technical skills (28), which can be attributed at the individual level to human factors (5, 6, 8). By practicing these skills in team situations, performance under stress may be improved (29).

### Do We Need More Real-Time Feedback?

Eye-tracking was used to measure visual attention, and Monivent provided real-time feedback in a recent randomized simulation study (10). Without the feedback, participants used chest rise and watched the position of the facemask to assess the effectiveness of ventilation. Real-time feedback was superior to using clinical assessment (10). In our study, we wanted to find out if prior training with real-time feedback improved simulation performance without feedback. We found that mask leakage, VT_*e*_, and ventilation rate were variable, perhaps because real-time feedback was not provided in the simulation. However, VT_*e*_ was often outside the recommended range, even with feedback. We speculate that students in the master’s degree program may need time to grow accustomed to feedback-devices while learning how to use them properly. For future research, it would be interesting to study whether the results improve with more training with real-time feedback before simulation. It might also be interesting to study whether training both with and without real-time feedback before simulation, improves performance.

### Strengths and Limitations

A strength of this study is that it contributes to filling a knowledge gap in our understanding of neonatal ventilation skills in nurses, as opposed to medical students and physicians. The study was a collaborative effort between the nursing and medical profession, and between higher education and clinical practice. Limitations include a low number of participants. Despite the low number of participants, the study indicates that human factors need more attention in the education of advanced clinical neonatal nurses. All the students had experience with simulation training in clinical practice before the start of the master’s degree program, but it is a limitation that this study did not include a baseline simulation, the reason for which has been explained in the methods section. The equipment we used, i.e., the self-inflating bag and mask, may not have been the best when a T-piece is often the device used in the clinical field. In further studies, the participants should be allowed to choose the resuscitation device they are most familiar with. The manikin was checked for an internal air leak, but it is still possible that this may have occurred undergoing ventilations.

Despite these limitations, we believe that our study provides useful learning points for educational contexts and clinical practice.

## Conclusion

For students in the master’s degree program in advanced clinical neonatal nursing, skills training with real-time feedback on mask leakage, tidal volume, ventilation rate, and inflation pressure did not result in high-quality bag-mask ventilation in a stressful simulated scenario. The objective measures agreed with the students’ own perceptions that their skills had not improved after the skills- and simulation training. In future studies, it would be of interest to try to distinguish individual differences influencing stress management in the performance of bag-mask ventilation in simulated neonatal resuscitation. It would also be interesting to investigate if more frequent training before simulation, and both blinded and visible real-time feedback training would improve the simulation results.

## Data Availability Statement

The raw data supporting the conclusions of this article will be made available by the authors, without undue reservation.

## Ethics Statement

The studies involving human participants were reviewed and approved by the Norwegian Center for Research Data (NSD)–reference number 890517. The patients/participants provided their written informed consent to participate in this study.

## Author Contributions

IR performed the data collection. IR and AS conceptualized the study and analyzed and interpreted the data. IR and A-KJ drafted the initial version of the manuscript. HA and AR contributed to the conceptualizing, data analysis, and interpretation. All authors participated in critical revision of the manuscript for important intellectual content, approved the final manuscript as submitted, and agreed to be accountable for all aspects of the work.

## Conflict of Interest

The authors declare that the research was conducted in the absence of any commercial or financial relationships that could be construed as a potential conflict of interest.

## Publisher’s Note

All claims expressed in this article are solely those of the authors and do not necessarily represent those of their affiliated organizations, or those of the publisher, the editors and the reviewers. Any product that may be evaluated in this article, or claim that may be made by its manufacturer, is not guaranteed or endorsed by the publisher.

## Abbreviations

VT_*e*_: expiratory tidal volume
NICU: neonatal intensive care unit
LDUC: Lovisenberg Diaconal University College
IQR: interquartile range
CPR: cardiopulmonary resuscitation.
